# Foot-and-Mouth Disease Virus VP3 Protein Acts as a Critical Proinflammatory Factor by Promoting Toll-Like Receptor 4-Mediated Signaling

**DOI:** 10.1128/JVI.01120-21

**Published:** 2021-11-09

**Authors:** Jing Zhang, Dan Li, Wenping Yang, Yue Wang, Lulu Li, Haixue Zheng

**Affiliations:** a State Key Laboratory of Veterinary Etiological Biology and OIE/National Foot and Mouth Disease Reference Laboratory, Lanzhou Veterinary Research Institute, Chinese Academy of Agricultural Sciences, Lanzhou, Gansu, China; University of California, Irvine

**Keywords:** foot-and-mouth disease virus, VP3, Rab7b, proinflammatory factor, TLR4

## Abstract

Foot-and-mouth disease virus (FMDV) infection in cloven-hoofed animals causes severe inflammatory symptoms, including blisters on the oral mucosa, hoof, and breast; however, the molecular mechanism underlying the inflammatory response is unclear. In this study, we provide the first evidence that the FMDV protein VP3 activates lipopolysaccharide-triggered Toll-like receptor 4 (TLR4) signaling. FMDV VP3 increased the expression of TLR4 by downregulating the expression of the lysozyme-related protein Rab7b. Additionally, Rab7b can interact with VP3 to promote the replication of FMDV. Our findings suggested that VP3 regulates the Rab7b-TLR4 axis to mediate the inflammatory response to FMDV.

**IMPORTANCE** Foot-and-mouth disease virus (FMDV) infection causes a severe inflammatory response in cloven-hoofed animals, such as pigs, cattle, and sheep, with typical clinical manifestations of high fever, numerous blisters on the oral mucosa, hoof, and breast, as well as myocarditis (tigroid heart). However, the mechanism underlying the inflammatory response caused by FMDV is enigmatic. In this study, we identified the VP3 protein of FMDV as an important proinflammatory factor. Mechanistically, VP3 interacted with TLR4 to promote TLR4 expression by inhibiting the expression of the lysozyme-related protein Rab7b. Our findings suggest that FMDV VP3 is a major proinflammatory factor in FMDV-infected hosts.

## INTRODUCTION

Foot-and-mouth disease virus (FMDV) belongs to the genus *Aphthovirus* of the family *Picornaviridae* and is a well-characterized pathogen affecting domestic and wild cloven-hoofed animals (1). FMDV is a single-stranded and positive-sense RNA virus with a genome of 8,500 nucleotides with a single open reading frame that encodes a polyprotein (2). This polyprotein is posttranslationally processed by virus-encoded proteases into four structural proteins, VP1 to VP4, and eight nonstructural proteins, L, 2A, 2B, 2C, 3A, 3B, 3C, and 3D (3). Mutations in FMDV confer the ability to evade hosts and counteract the complex host innate immune response (4). However, the mechanism by which FMDV regulates the Toll-like receptor 4 (TLR4) signaling pathway in animal hosts is still unclear.

TLRs play important roles in both innate and adaptive immune responses (5). TLRs contain an extracellular leucine-rich repeat (LRR) domain, which recognizes a distinct set of pathogen-associated molecular patterns (PAMPs), and an intracellular signaling Toll-IL-1 receptor (TIR) domain, which is conserved among all Toll and interleukin-1 receptors (6, 7). The TIR domain is responsible for homotypic protein–protein interactions and recruits downstream TIR domain-containing adaptor proteins, such as myeloid differentiation primary-response gene 88 (MyD88) and the TIR domain-containing adaptor inducing beta interferon (IFN-β) (TRIF; also called TICAM-1) (7). The MyD88-dependent or TRIF-dependent signaling cascade activates several transcription factors, leading to the induction of proinflammatory cytokines and type I IFNs (7–9). All TLRs, at least to some extent, could trigger signal transduction via the MyD88-IRAK4-IRAK1/2-TRAF6-IKK axis to activate the NF-κB pathway (10–12). TLR4, which recognizes lipopolysaccharides (LPS) in Gram-negative bacteria, is the only receptor that functions via an MyD88-dependent pathway to activate NF-κB- and TRIF-dependent pathways, thereby upregulating both the NF-κB and IRF3 signaling pathways (13).

In this study, we identified the FMDV protein VP3 as a potent activator of TLR4 signaling. The protein is involved in the type I and type II IFN signaling pathways (14, 15). However, the role of VP3 in relation to the TLR4 signaling pathway has not been determined. We demonstrated that FMDV VP3 interacts with TLR4 to promote TLR4 expression by downregulating Rab7b expression. Our findings suggested that the TLR4–Rab7b axis mediates the host inflammatory response against FMDV and that FMDV VP3 is a crucial proinflammatory factor.

## RESULTS

### FMDV positively regulates the LPS-induced transcription of downstream genes.

**FIG 1:**
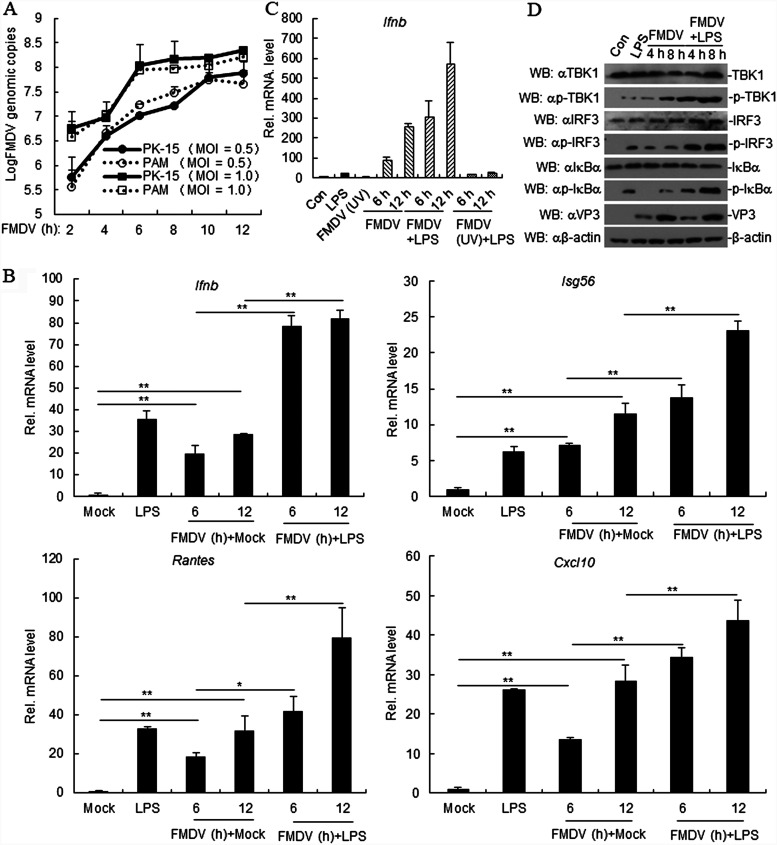
Foot-and-mouth virus (FMDV) potentiates the lipopolysaccharide (LPS)-induced signaling pathway. (A) PAMs are susceptible to FMDV. PK-15 and PAMs were seeded in a 12-well plate for 12 h. The cells then were infected with FMDV (MOI, 0.5 or 1.0) for the indicated times before RT-PCR experiments. (B) Effects of FMDV on LPS-induced increases in *Ifnb*, *Isg56*, *Rantes*, and *Cxcl-10* in porcine alveolar macrophages (PAMs). PAMs were seeded in a 12-well plate for 12 h. The cells then were uninfected or infected with FMDV (MOI, 1.0) for the indicated times. Cells were treated or left untreated with LPS (1 μg/ml) for 4 h before RT-PCR experiments. (C) Inactivated FMDV did not induce the transcription of *Ifnb* in PAMs. After PAMs were seeded in a 12-well plate for 12 h, cells were uninfected or infected with FMDV or inactive-FMDV (obtained by UV treatment for 30 min) (MOI, 1.0) for the indicated time. The cells were treated or left untreated with LPS (1 μg/ml) for 4 h before RT-PCR. (D) FMDV increases the LPS-induced phosphorylation of TBK1, IRF3, and IκBα. PAMs were seeded in a 12-well plate for 24 h. Cells then were uninfected or infected with FMDV (MOI, 1.0) for the indicated times. Cells were left untreated or treated with LPS (1 μg/ml) for 4 h. Cell lysates were analyzed by immunoblotting with the indicated antibodies. Data are shown as means ± SD. Data are representative of at least three independent experiments. RT-PCR, real-time PCR; SD, standard deviation; Con, control; EV, empty vector; WB, Western blot.

Porcine alveolar macrophages (PAMs) and porcine kidney (PK-15) cells were infected with FMDV to evaluate susceptibility. The FMDV genome copy number was lower in PAMs than in PK-15 cells, although the difference was not significant (Fig. 1A), suggesting that PAMs are susceptible to FMDV. To determine the effect of FMDV on LPS-triggered signaling pathways, PAMs were infected with FMDV, followed by treatment with LPS. As determined by real-time PCR (RT-PCR), transcript levels of *Isg56*, *Cxcl10*, *Ifnb*, and *Rantes* were higher in PAMs infected with FMDV than in uninfected cells (Fig. 1B), suggesting that the LPS-mediated signaling pathway is upregulated post-FMDV infection. To exclude the background effect of the production of FMDV particles or the presence of foreign RNA on levels of transcription, we evaluated LPS-induced *IFN-β* expression in UV-irradiated FMDV-infected PAMs. LPS, FMDV, and LPS-treated FMDV induced *IFN-β* transcription in PAMs, while UV-treated FMDV had no effect (Fig. 1C). In addition, FMDV infection resulted in increased activity of LPS-triggered TBK1, IRF3, and IκBα phosphorylation compared to levels in control cells (Fig. 1D). Our findings suggested that FMDV positively regulates the LPS-triggered signaling pathway.

### FMDV VP3 is a positive regulator of TLR4 signaling.

**FIG 2:**
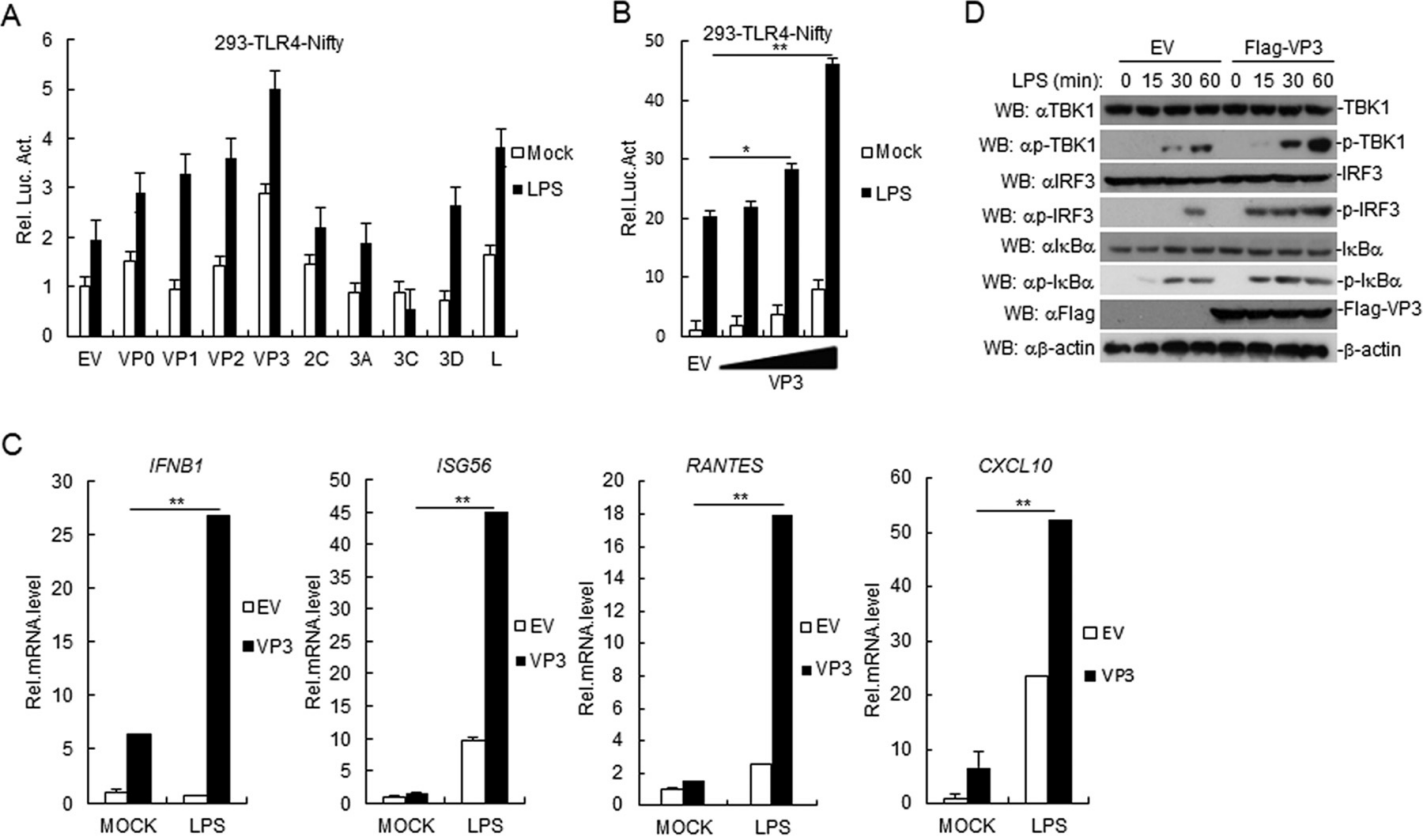
FMDV VP3 regulates the TLR4-mediated signaling pathway. (A) Effect of the overexpression of FMDV proteins on LPS-triggered activation of the Nifty promoter. 293-TLR4 cells (1 × 10^5^) were transfected with the Nifty reporter (0.1 μg) and the indicated expression plasmids. Twenty hours after transfection, cells were treated or left untreated with LPS for 12 h before luciferase assays. Rel. Luc. Act., relative luciferase activity. (B) Dose-dependent effects of FMDV VP3 on LPS-triggered activation of the Nifty promoter. The experiments were performed following similar methods to those in panel A. (C) Effects of FMDV VP3 on the LPS-induced increases in *Ifnb*, *Isg56*, *Rantes*, and *Cxcl-10*. VP3-overexpessing PAMs (2 × 10^5^) were left untreated or treated with LPS (1 μg/ml) for 12 h before RT-PCR experiments were performed with the indicated primers. (D) FMDV VP3 increases LPS-induced phosphorylation of TBK1, IRF3, and IκBα. FMDV VP3-overexpressed PAM cells were left untreated (2 × 10^5^) or were treated with LPS (1 μg/ml) for the indicated times. Cell lysates were analyzed by immunoblotting with the indicated antibodies. Data are shown as means ± SD. Data are representative of at least three independent experiments. RT-PCR, real-time PCR; SD, standard deviation; EV, empty vector.

To identify FMDV proteins that increase LPS-mediated induction of downstream antiviral genes, we first constructed nine expression clones encoding individual FMDV proteins. Those clones were further screened by reporter assays for their ability to regulate the activation of Nifty (an NF-κB promoter) in 293-TLR4 cells. Among the candidate clones that markedly increased LPS-mediated Nifty activation, FMDV VP3 exerted the strongest effect (Fig. 2A). Based on reporter assays, we found that ectopic expression of FMDV VP3 in 293-TLR4 cells increased LPS-induced activation of Nifty in a dose-dependent manner (Fig. 2B). RT-PCR analysis indicated that ectopic expression of FMDV VP3 increased LPS-induced transcription of *Isg56*, *Cxcl10*, *Ifnb*, and *Rantes* in PAMs (Fig. 2C). In addition, ectopic expression of FMDV VP3 increased the phosphorylation of TBK1, IRF3, and IκBα induced by LPS in PAMs (Fig. 2D), suggesting that FMDV VP3 increased LPS-triggered downstream signaling events. Our results suggested that FMDV VP3 increases the LPS-triggered induction of downstream antiviral genes.

### FMDV VP3 regulates TLR4 signaling at the TLR4 level.

**FIG 3:**
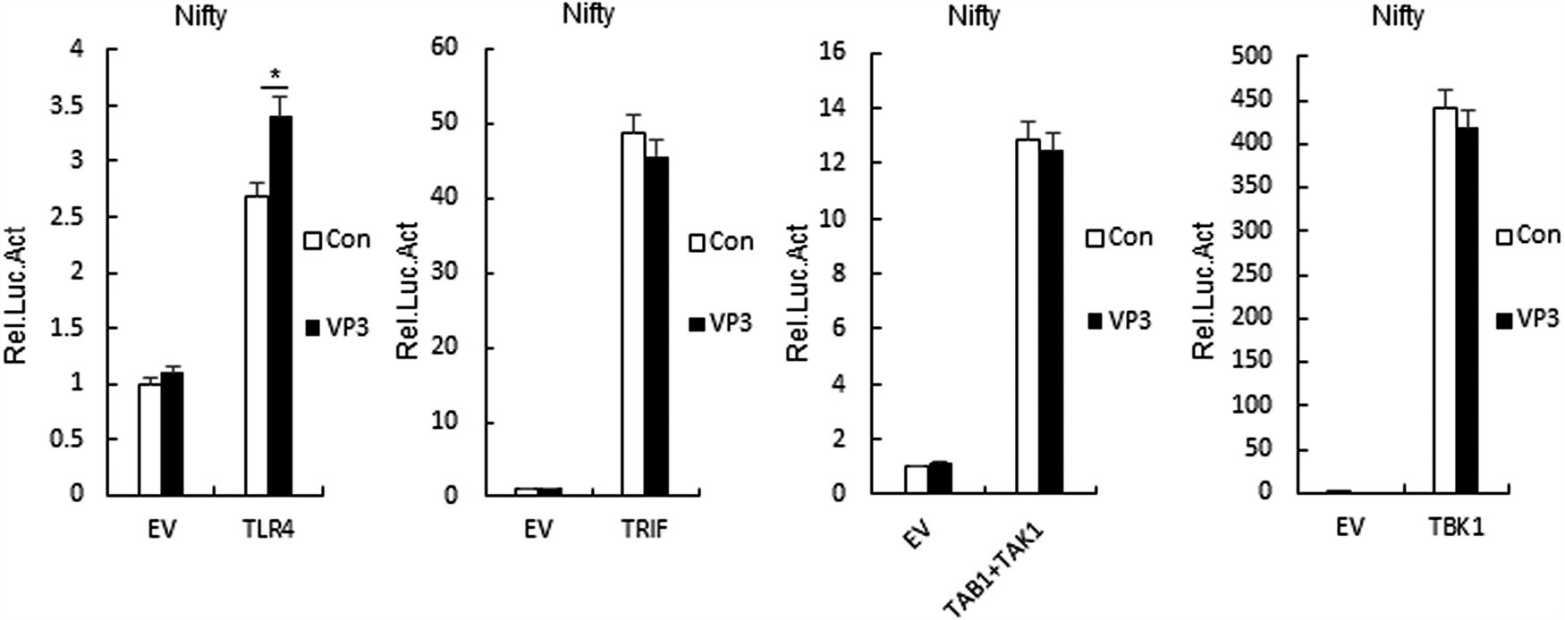
FMDV VP3 regulates the TLR4 signaling pathway at the TLR4 level. 293-TLR4 cells (1 × 10^5^) were transfected with the Nifty reporter (0.1 μg) and the indicated expression (0.1 μg) plasmids for 24 h before luciferase assays. Data are shown as means ± SD. Data are representative of at least three independent experiments. SD, standard deviation; Luc, luciferase. EV, empty vector; Con, control.

Various components are involved in TLR4 signaling pathway. As shown in Fig. 3, FMDV VP3 increased Nifty activation via TLR4 but not via TRIF, TAK1 and TAB1, and TBK1. These results suggested that FMDV VP3 targets TLR4.

### FMDV VP3 interacts with TLR4.

**FIG 4:**
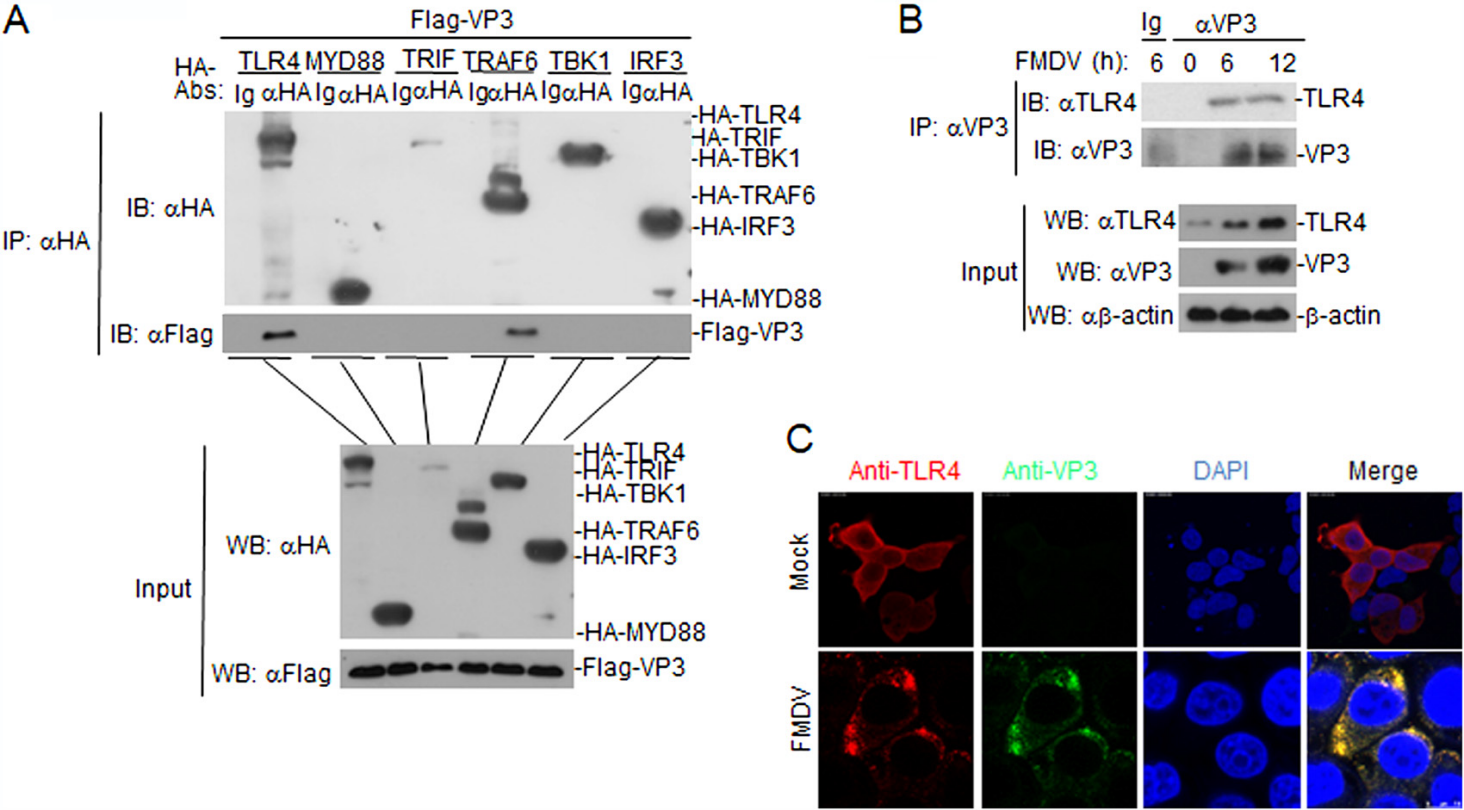
FMDV VP3 interacts with TLR4. (A) FMDV VP3 interacted with TLR4 and TRAF6 but not with Myd88, TRIF, TBK1, or IRF3 in the overexpression system. 293T cells (2 × 10^6^) were transfected with the indicated plasmids (5 μg each). Coimmunoprecipitation and immunoblot analyses were performed with the indicated antibodies. The expression of transfected proteins was analyzed by immunoblotting with anti-HA or anti-Flag antibodies. (B) Endogenous interactions between VP3 and TLR4. PAMs were left uninfected or were infected with FMDV for the indicated times before coimmunoprecipitation and immunoblotting. (C) Colocalization of VP3 with endogenous TLR4. PAMs were mock infected or infected with FMDV. Cells were fixed at 12 h postinfection and subjected to indirect immunofluorescence to detect VP3 (green) and TLR4 (red) with mouse anti-VP3 and rabbit anti-TLR4 antibodies. Nuclei are indicated by 4′,6-diamidino-2-phenylindole (DAPI) (blue) staining in the merged image.

We next investigated whether FMDV VP3 could interact with TLR4. Transient-transfection and coimmunoprecipitation (co-IP) experiments indicated that FMDV VP3 was associated with TLR4 and TRAF6 but not with Myd88, TRIF, TBK1, and IRF3 (Fig. 4A). To determine whether FMDV VP3 interacts with cellular TLR4 in the context of FMDV infection, virus-infected PAMs lysates were immunoprecipitated with a mouse anti-VP3 polyclonal antibody and probed for the presence of TLR4 with a rabbit anti-TLR4 polyclonal antibody. TLR4 was readily detected in FMDV-infected PAMs (Fig. 4B), indicating that TLR4 could interact with endogenous VP3 in FMDV-infected PAMs. To confirm that the colocation was due to the interaction between endogenous TLR4 and VP3, PAMs were infected with FMDV for 12 h and examined by confocal microscopy. Confocal images of cells immunostained with anti-VP3 and anti-TLR4 antibodies confirmed the colocalization of TLR4 with FMDV VP3 (Fig. 4C). Collectively, these findings confirmed that TLR4 could interact with the FMDV VP3 protein.

### TLR4 inhibits FMDV replication.

**FIG 5:**
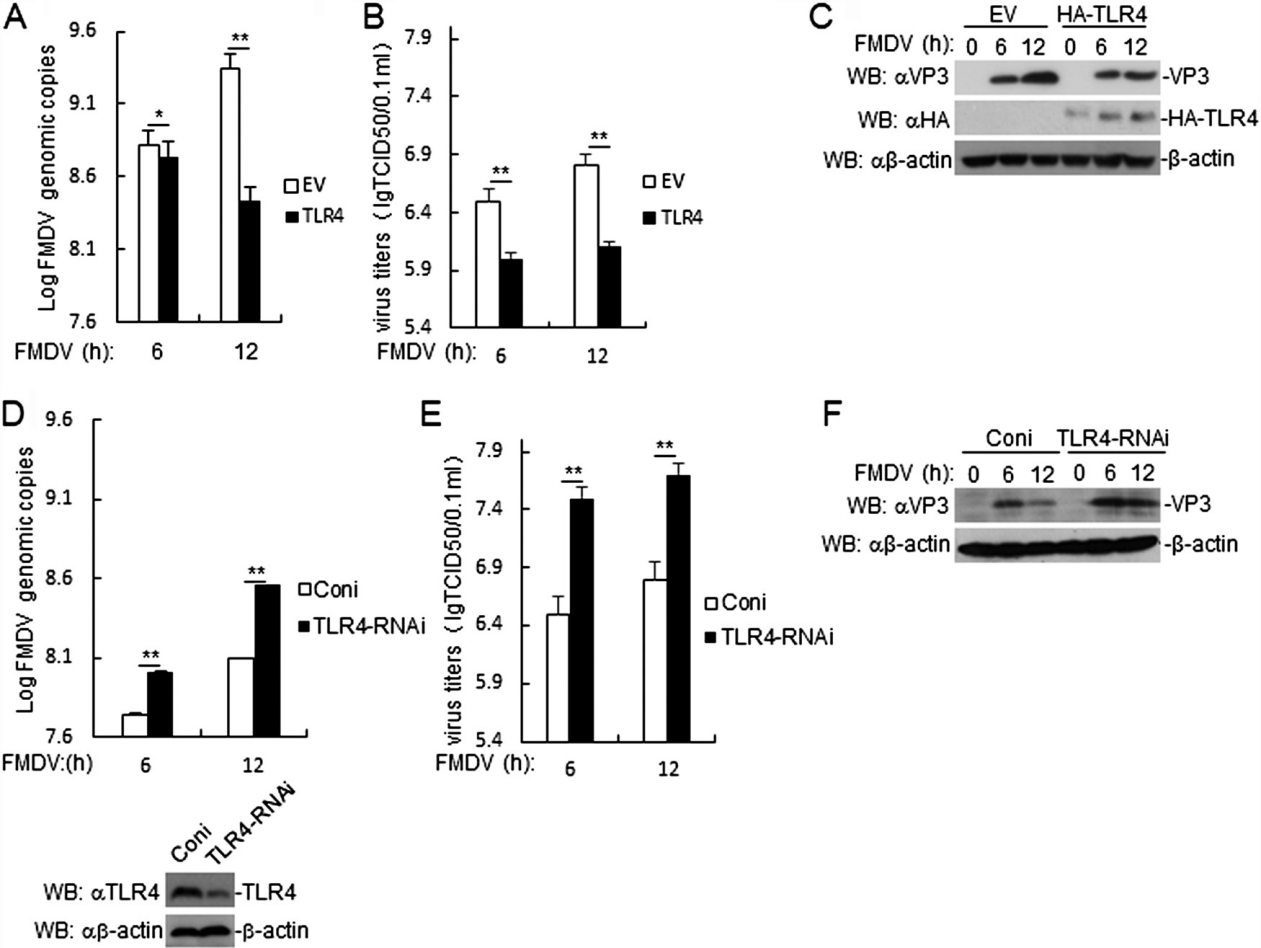
TLR4 inhibits FMDV replication. (A) Effects of TLR4 overexpression in PAMs on FMDV replication. TLR4-overexpressing PAMs were infected with FMDV (MOI, 1.0) for the indicated times. FMDV genome replication was evaluated by RT-PCR. The results are representative of three independent experiments, and means ± SD from triplicate assays are shown. (B) Effects of TLR4 overexpression on FMDV titers in PAM cells. TLR4-overexpressing PAMs were infected with FMDV (MOI, 1.0) for the indicated times. The virus titers in the supernatants were determined by immunofluorescence and are expressed as 50% tissue culture infective dose (TCID_50_)/0.1 ml. The results are representative of three independent experiments, and means ± SD are shown. (C) Effects of TLR4 overexpression on FMDV protein expression in PAMs. TLR4-overexpressing PAMs were infected with FMDV (MOI, 1.0) for the indicated times. FMDV VP3 was detected by immunoblotting with the indicated antibodies. (D) Effects of TLR4 knockdown in PAMs on FMDV replication. The experiments were performed by following methods similar to those for panel A. (E) Effects of TLR4 knockdown on FMDV titers in PAMs. The experiments were performed by following methods similar to those of panel B. (F) Effects of TLR4 knockdown on FMDV protein expression in PAMs. The methods were similar to those for panel C. Data are shown as means ± SD. Data are representative of at least three independent experiments. EV, empty vector; Coni, control RNAi.

To determine the role of TLR4 in FMDV replication, TLR4-overexpressing PAMs were infected with FMDV. Based on RT-PCR and viral titer experiments, we found that TLR4-overexpressing PAMs inhibited the replication of FMDV (Fig. 5A and B). Similarly, the expression of FMDV VP3 was inhibited in TLR4-overexpressing PAMs (Fig. 5C). We then constructed a porcine TLR4-RNA interference plasmid, and an immunoblot analysis indicated that this plasmid markedly inhibited the expression of endogenous TLR4 in PAMs (Fig. 5D). RT-PCR and viral titer experiments showed that FMDV replication was higher in TLR4-knockdown PAMs cells than in control cells (Fig. 5D and E). Furthermore, we found that the expression level of FMDV VP3 was higher in TLR4-knockdown PAMs than in control cells (Fig. 5F). These results suggested that TLR4 attenuated the replication of FMDV.

### FMDV increases TLR4 expression via the lysozyme pathway.

**FIG 6:**
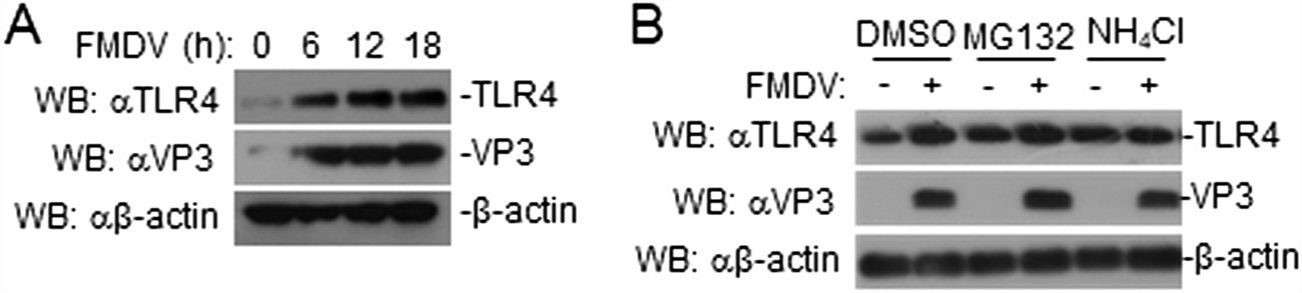
FMDV potentiates TLR4 via the lysozyme pathway. (A) Effects of FMDV on endogenous TLR4 in PAMs. Briefly, PAMs were uninfected or infected with FMDV for the indicated times and then analyzed by immunoblotting with the indicated antibodies. (B) Effects of inhibitors on the FMDV-mediated stability of TLR4. PAMs were treated with the indicated inhibitors for 1 h. The cells then were left uninfected or infected with FMDV for 6 h and were analyzed by immunoblotting with the indicated antibodies. DMSO, dimethyl sulfoxide.

To explore the effect of FMDV infection on TLR4 expression, PAMs were infected with FMDV at the indicated times. FMDV infection increased the expression of TLR4 (Fig. 6A). To investigate the mechanisms by which FMDV affects the stability of TLR4, we treated cells with various inhibitors of protein degradation pathways. The lysosome inhibitor ammonium chloride (NH_4_Cl), but not a proteasome inhibitor (MG132), blocked the increase in TLR4 in FMDV-infected PAMs (Fig. 6B). These results suggested that FMDV increases the expression of TLR4 via the lysozyme pathway.

### FMDV VP3 maintains the stability of TLR4 by downregulating Rab7b expression.

**FIG 7:**
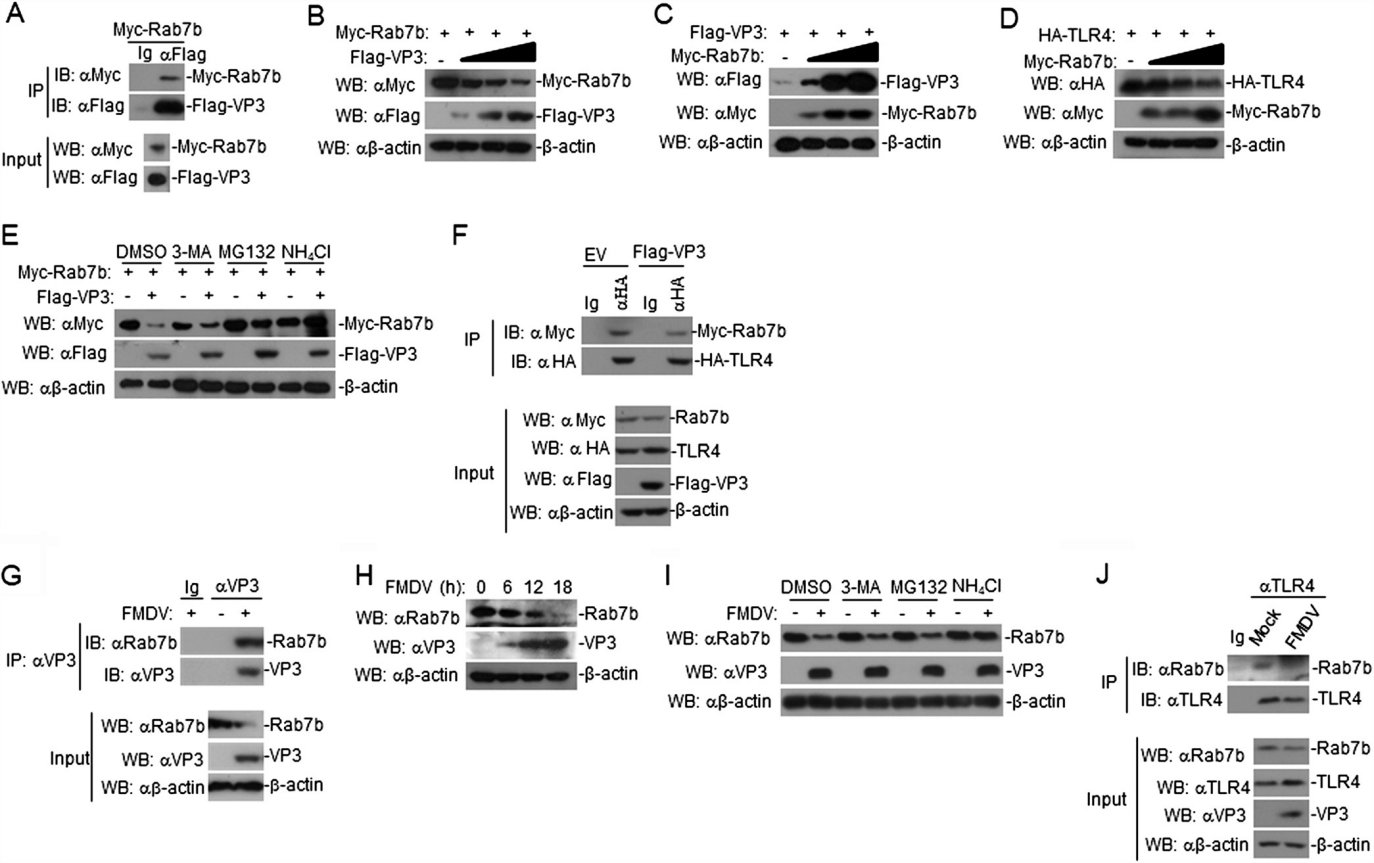
FMDV VP3 potentiated TLR4 expression by downregulating Rab7b expression. (A) FMDV VP3 interacted with Rab7b in the overexpression system. 293T cells (2 × 10^6^) were transfected with the indicated plasmids (5 μg each). Coimmunoprecipitation and immunoblot analyses were performed with the indicated antibodies. The expression of transfected proteins was analyzed by immunoblotting with anti-Myc or anti-Flag antibodies. (B) VP3 inhibits the expression of Rab7b protein. 293T cells (2 × 10^5^) were transfected with Myc-Rab7b (1 μg) and Flag-VP3 (0 μg, 0.25 μg, 0.5 μg, and 1.0 μg) for 24 h. Cell lysates were analyzed by immunoblotting with the indicated antibodies. (C) Rab7b increases the expression of VP3. The methods were similar to those for panel B. (D) Rab7b inhibits the expression of TLR4. The methods were similar to those for panel B. (E) Effects of inhibitors on the VP3-mediated stabilization of Rab7b. 293T cells (4 × 10^5^) were transfected with the indicated plasmids. Eighteen hours after transfection, the cells were treated with the indicated inhibitors for 6 h before immunoblot analyses. (F) Effects of FMDV VP3 on the interaction between TLR4 and Rab7b. The methods were similar to those for panel A. (G) Endogenous associations between VP3 and Rab7b. PAMs were left uninfected or were infected with FMDV for 12 h before coimmunoprecipitation and immunoblot analysis. (H) Effects of FMDV on endogenous Rab7b in PAMs. Briefly, PAMs were left uninfected or were infected with FMDV for the indicated times and then analyzed by immunoblotting with the indicated antibodies. (I) Effects of inhibitors on the FMDV-mediated stabilization of Rab7b. PAMs were treated with the indicated inhibitors for 1 h. The cells then were left uninfected or were infected with FMDV for 6 h and analyzed by immunoblotting with the indicated antibodies. (J) FMDV inhibits the interaction between TLR4 and Rab7b in PAMs. The methods were similar to those for panel G.

A previous study has shown that TLR4 interacts with Rab7b (16), prompting us to investigate whether Rab7b-mediated FMDV VP3 had an effect on TLR4. To determine whether FMDV VP3 is associated with Rab7b, co-IP experiments were performed using 293T cells transiently coexpressing Flag-tagged FMDV VP3 and Myc-tagged Rab7b plasmids. The results showed that FMDV VP3 interacted with Rab7b (Fig. 7A). By the co-IP of endogenous proteins, we found that FMDV VP3 was associated with Rab7b in FMDV-infected PAMs (Fig. 7G). Additionally, FMDV VP3 inhibited the expression of Rab7b in a dose-dependent manner in 293T cells (Fig. 7B). We also found that FMDV consistently inhibited the expression of Rab7b in PAMs (Fig. 7H). Further experiments indicated that Rab7b increased the expression of FMDV VP3 and inhibited the expression of TLR4 in a dose-dependent manner in 293T cells (Fig. 7C and D). Moreover, we found that NH_4_Cl, but neither MG132 nor autophagosome inhibitor 3-methyladenine (3-MA), inhibited the degradation of Rab7b in VP3-overexpressing 293T cells (Fig. 7E). Consistent with this, NH_4_Cl, but not MG132 or 3-MA, blocked the degradation of Rab7b in FMDV-infected PAMs (Fig. 7I). To confirm that FMDV VP3 influences the interaction between TLR4 and Rab7b, 293T cells were transfected with TLR4 and Rab7b or FMDV VP3. Co-IP experiments showed that FMDV VP3 inhibited the interaction between TLR4 and Rab7b in 293T cells (Fig. 7F). Consistent with these findings, FMDV also disrupted the interaction between TLR4 and Rab7b in FMDV-infected PAMs (Fig. 7J). Our results demonstrated that FMDV VP3 maintained the stability of TLR4 by downregulating Rab7b expression.

### Rab7b expression promotes FMDV replication.

**FIG 8:**
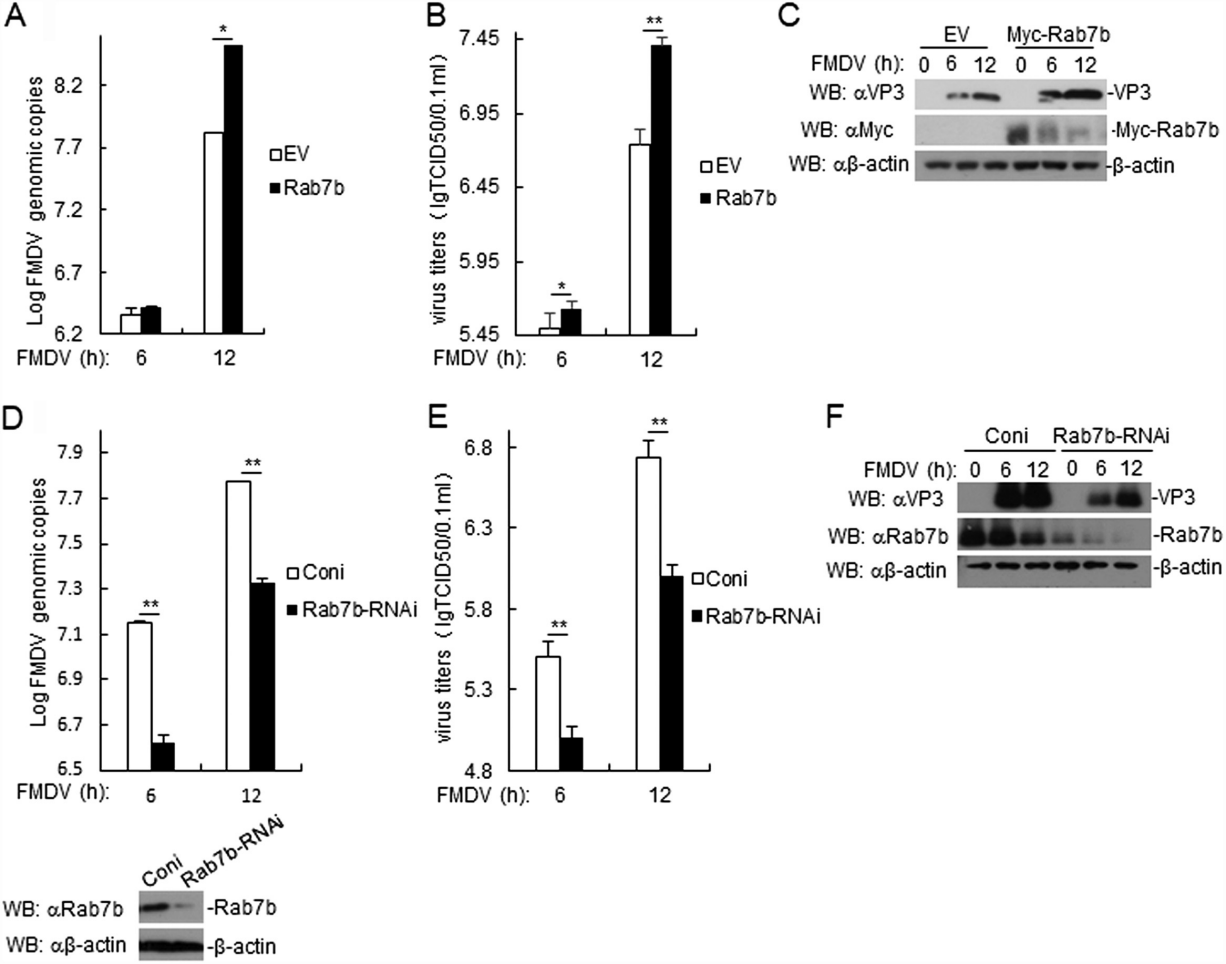
Rab7b overexpression promotes FMDV replication. (A) Effects of Rab7b overexpression in PAMs on FMDV replication. Rab7b-overexpressed PAMs were infected with FMDV (MOI, 1.0) for the indicated times. FMDV genome replication was evaluated by RT-PCR. The results are representative of three independent experiments, and means ± SD are shown. (B) Effect of Rab7b overexpression on FMDV titers in PAMs. Rab7b-overexpressed PAMs were infected with FMDV (MOI, 1.0) for the indicated times. The virus titers in the supernatants were determined by immunofluorescence and are expressed as TCID_50_/0.1 ml. The results are representative of three independent experiments and are presented as means ± SD from three technical replicates. (C) Effect of Rab7b overexpression on FMDV protein expression in PAMs. Rab7b-overexpressing PAMs were infected with FMDV (MOI, 1.0) for the indicated times. FMDV VP3 protein expression was detected by immunoblotting with the indicated antibodies. (D) Effect of Rab7b knockdown in PAMs on FMDV replication. The methods were similar to those for panel A. (E) Effect of Rab7b knockdown on FMDV titers in PAMs. The methods were similar to those for panel B. (F) Effect of Rab7b knockdown on FMDV protein expression in PAMs. The methods were similar to those for panel C.

To examine whether Rab7b plays a role in RNA synthesis in FMDV, Rab7b-overexpressing PAMs were infected with FMDV. The genome copy number of FMDV RNA in Rab7b-overexpressing PAMs was higher than that in control cells (Fig. 8A). We observed higher FMDV titers in the supernatants of Rab7b-overexpressing PAMs than in control cells (Fig. 8B). In addition, we found that Rab7b-overexpressing PAMs exhibited an increase in the expression of FMDV VP3 after FMDV infection (Fig. 8C). Conversely, PAMs with Rab7b knockdown exhibited the opposite trend (Fig. 8D to F). Taken together, our results suggested that Rab7b increases FMDV replication.

## DISCUSSION

The ability of viruses to evade and modulate the host immune response plays an important role in the successful establishment and persistence of infection. The innate immune system constitutes the first line of defense against infection (17). Therefore, viruses have acquired sophisticated strategies to inhibit and evade the innate immune system, and knowledge of these strategies will substantially improve our understanding of the pathogenesis of viral diseases (18). In this study, we employed various expression screening methods and identified FMDV VP3 as a positive regulator of the LPS-mediated induction of downstream antiviral genes and the innate antiviral response.

We obtained substantial evidence suggesting that FMDV VP3 directly targets TLR4 to modulate the innate antiviral response. First, FMDV VP3 is closely associated with TLR4. Second, the overexpression of FMDV VP3 increased TLR4- but not the TRIF-, TAK1- and TAB1-, and TBK1-mediated induction of downstream antiviral genes in 293-TLR4 cells.

In FMDV serotype O, the VP3 protein comprises 220 amino acid residues and is the most conserved of all the structural proteins of picornaviruses. Previous studies have suggested that FMDV VP3 inhibits the type I IFN signaling pathway by disrupting *VISA* mRNA and the type II IFN signaling pathway by degrading JAK1 (14, 15). In this study, we observed that FMDV could elicit inflammation in PAMs. However, the mechanism underlying the proinflammatory response has not been determined. TLR4 is a transmembrane protein expressed mainly in macrophages and plays an important role in recognizing and mediating macrophage activation and proinflammatory cytokine release (19). Our results demonstrated that FMDV VP3 interacts with TLR4 and could activate the TLR4 signaling pathway. TLR4 signaling results in a potent inflammatory response aimed at eliminating the invading pathogen (20). Overexpression of TLR4 inhibited FMDV replication. During an acute infection by FMDV, not only FMDV but also host proteins, such as TLR4, could inhibit IFN production and induce an inflammatory response, resulting in competition between the host and pathogen for survival. The inflammatory response, induced by VP3 or other FMDV proteins, is detrimental to the host and makes the virus a more effective pathogen. In addition, VP3 or other FMDV proteins also increase TLR4 expression, thereby inhibiting viral replication. The virus benefits from a symbiotic relationship with the host rather than by destroying the host.

Rab7b is a negative regulator of TLR4 signaling by interfering with its trafficking. It acts as a novel lysosome-associated small GTPase to negatively regulate TLR4 signaling by promoting its lysosomal degradation (21, 22). We obtained multiple lines of evidence suggesting that Rab7b is involved in the FMDV VP3-mediated TLR4 signaling pathway. First, VP3 is associated with Rab7b. Second, FMDV or VP3 could inhibit the expression of Rab7b. Third, Rab7b increased FMDV replication in PAMs.

Previous studies have demonstrated that dominant-negative mutants of Rab7 have no effect on FMDV-infected IBRS-2 cells (23). IBRS-2 is a nonimmune cell line that does not express TLR4. In this study, we examined the effect of Rab7b on FMDV replication by PAMs, which can express TLR4.

Based on our findings and previous results, we propose a working model of the FMDV VP3-mediated regulation of the TLR4 signaling pathway. During the course of infection by FMDV, VP3 interacts with Rab7b and TLR4 and subsequently attenuates the expression of Rab7b, which results in the upregulation of TLR4 expression. Our results provide a crucial theoretical basis for the mechanism underlying the FMDV-elicited inflammatory response.

## MATERIALS AND METHODS

### Cell lines, viruses, and antibodies.

Human embryonic kidney 293T (293T) cells were purchased from the ATCC. 293-TLR4 cells were kindly provided by Hong-Bing Shu (Wuhan University, China). PAMs were prepared according to a published protocol (24), and alveolar macrophages were isolated using bronchoalveolar lavage and grown in Dulbecco’s modified Eagle’s medium (DMEM) supplemented with 2 mM l-glutamine, 100 U/ml gentamicin, nonessential amino acids, and 10% porcine serum. Cells were grown at 37°C in an incubator with 5% CO_2_ saturated with water vapor. Rabbit polyclonal antibodies against TBK1, p-TBK1, IRF3, p-IRF3, IκBα, p-IκBα, TLR4, and Rab7b were purchased from Cell Signaling Technology (Danvers, MA, USA). Mouse monoclonal antibodies against Flag, Myc, IgG (Ig), β-actin (Sigma, St. Louis, MO, USA), and hemagglutinin (HA; OriGene, Rockville, MD, USA) were obtained. Mouse anti-VP3 sera were prepared in our laboratory using a recombinant FMDV VP3 protein. Type O FMDV was propagated in PK-15 cells, and the supernatants were clarified and stored at −80°C.

### Plasmid construction.

A mammalian expression plasmid for Myc-Rab7b was constructed by standard molecular biology techniques. FMDV expression plasmids were constructed as described previously (25). In addition, the Nifty promoter luciferase reporter plasmids, pRL-TK plasmid, and mammalian expression plasmids for HA-tagged TLR4, TRIF, TAK1, TAB1, TBK1, Myd88, TRAF6, and IRF3 were constructed as previously described (26, 27).

### Transfection and reporter gene assays.

293-TLR4 cells (1 × 10^5^) were seeded on 48-well plates and transfected the following day via standard calcium phosphate precipitation. An empty control plasmid was added to ensure that each transfection receives the same amount of total DNA. Here, 0.01 μg of pRL-TK *Renilla* luciferase reporter plasmid was added to each transfection to normalize the transfection efficiency. Luciferase assays were performed using a dual-specific Luciferase assay kit (Promega, Madison, WI, USA), and firefly luciferase activity levels were normalized against *Renilla* luciferase activity.

### RNA interference.

Double-stranded oligonucleotides corresponding to the target sequences were cloned into the lentiCRISPR-V2 vector and cotransfected with packaging plasmids into 293T cells. Lentiviral particles were collected and used to transduce PAMs. The infected PAMs were selected with puromycin (1 μg/ml) for 2 weeks before additional experiments were performed. The following sequences were targeted: porcine *TLR4* cDNA, GCCAGGACGAAGACTGGGTG; porcine *Rab7b* cDNA, ATGATCTTAGAGAGGATGC.

### Co-IP and immunoblot analyses.

Co-IP and immunoblot experiments were performed as previously described (28–33). For transient-transfection and co-IP experiments, HEK293T cells were transfected with the appropriate plasmid. Twenty-four hours later, the cells were harvested and lysed in 1 ml of lysis buffer (20 mM Tris, pH 7.5, 150 mM NaCl, 1% Triton, 1 mM EDTA, 10 μg/ml aprotinin, 10 μg/ml leupeptin, and 1 mM phenylmethylsulfonyl fluoride). For each sample, 0.4 ml of cell lysate was incubated with 0.5 μg of the indicated antibody or control IgG and 40 μl of protein G agarose beads (Santa Cruz Biotechnology, Inc., Houston, TX, USA) at 4°C. After 4 h of incubation, the beads were washed three times with 1 ml of lysis buffer containing 0.5 M NaCl. The precipitates were analyzed by immunoblotting.

For endogenous co-IP experiments, PAMs (5 × 10^7^) were infected with FMDV for the indicated time periods. Co-IP and immunoblot experiments were performed as previously described.

### Overexpression-transduced stable PAMs.

293T cells were transfected with two packaging plasmids (pGAG-Pol and pVSV-G) combined with a control or TLR4, Rab7b, or VP3 overexpression retroviral plasmid. Twenty-four hours later, the cells were incubated with fresh medium without antibiotics for another 24 h. The recombinant virus-containing medium was filtered and added to PAMs in the presence of Polybrene (8 μg/ml). The infected cells were selected with puromycin (0.5 μg/ml) for 7 days before additional experiments.

### FMDV titer assay.

The FMDV titers in the collected supernatants were determined by a virus titration assay, as previously described (34). Briefly, a BHK-21 cell suspension in DMEM with 5% FBS at a concentration of 1.5 × 10^6^ cells/ml was dispensed at 50 μl per well into 96-well flat-bottomed tissue culture plates. The plates were rocked to achieve a uniform suspension thickness and incubated at 37°C for 24 h to 36 h under 5% CO_2_ to attain 90% confluence. Serial 10-fold dilutions of the virus stock prepared in FBS-free DMEM were added in 50-μl volumes to all wells. Plates were incubated at 37°C, 5% CO_2_ for 72 h, and the presence or absence of a cytopathic effect (CPE) was then monitored. FMDV titers were calculated by the Reed-Muench method.

### RT-PCR analysis.

PAMs were treated with LPS or left untreated and infected with FMDV at a multiplicity of infection (MOI) of 0.1, or VP3-overexpressing PAMs were treated with LPS or left untreated. Subsequently, total RNA was isolated from cells by using TRIzol reagent (TaKaRa, Kusatsu, Japan) and subjected to real-time PCR to evaluate the FMDV genome copies using iQ SYBR green Supermix and a C1000 Thermal Cycler (Bio-Rad, Hercules, CA, USA). Gene-specific primer sequences were the following: *Ifnb*, 5′-CACTGGCTGGAATGAAACCG-3′, 5′-AATGGTCATGTCTCCCCTGG-3′; *Rantes*, 5′-ACACCCTGCTGTTTTTCCTACCT-3′, 5′-AGACGACTGCTGCCATGGA-3′; *Cxcl10*, 5′-GGTGAGAAGAGATGTCTGAATCC-3′, 5′-GTCCATCCTTGGAAGCACTGCA-3′; *Isg56*, 5′-TCATCAGGTCAAGGATAGTC-3′, 5′-CCACACTGTATTTGGTGTCTAGG-3′; *Gapdh*, 5′-GAGTCAACGGATTTGGTCGT-3′, 5′-GACAAGCTTCCCGTTCTCAG-3′.

Genome copy numbers were quantified by following previously described quantitative RT-PCR methods (35, 36).

### Statistical analysis.

Samples were compared using an unpaired two-tailed Student's *t* test. Means and standard deviations (SD) are shown in figures. All experiments were performed independently at least three times, and representative results are shown.
